# Dimensions of Animal Consciousness

**DOI:** 10.1016/j.tics.2020.07.007

**Published:** 2020-08-20

**Authors:** Jonathan Birch, Alexandra K. Schnell, Nicola S. Clayton

**Affiliations:** 1 Centre for Philosophy of Natural and Social Science, London School of Economics and Political Science, Houghton Street, London, WC2A 2AE, UK; 2 Comparative Cognition Lab, Department of Psychology, University of Cambridge, Cambridge CB2 3EB, UK

## Abstract

How does consciousness vary across the animal kingdom? Are some animals ‘more conscious’ than others? This article presents a multidimensional framework for understanding interspecies variation in states of consciousness. The framework distinguishes five key dimensions of variation: perceptual richness, evaluative richness, integration at a time, integration across time, and selfconsciousness. For each dimension, existing experiments that bear on it are reviewed and future experiments are suggested. By assessing a given species against each dimension, we can construct a consciousness profile for that species. On this framework, there is no single scale along which species can be ranked as more or less conscious. Rather, each species has its own distinctive consciousness profile.

## The Emerging Science of Animal Consciousness

A conscious being has subjective experiences of the world and its own body. Humans are conscious beings, but are we alone? In 2012, the Cambridge Declaration on Consciousness crystallised a scientific consensus that humans are not the only conscious beings and that ‘non-human animals, including all mammals and birds, and many other creatures, including octopuses’ possess neurological substrates complex enough to support conscious experiences. This consensus has allowed debates about animal consciousness to move on from the old question of whether any non-human animals are conscious to the questions of which animals are conscious and what form their conscious experiences take.

In the past 5 years, an interdisciplinary community of animal consciousness researchers, drawn from neuroscience, evolutionary biology, comparative psychology, animal welfare science, and philosophy, has begun to coalesce around these questions, as shown, for example, by the founding of the journal *Animal Sentience* in 2016 [1]. The aim of this field is to study the inner lives of animals (their subjective experiences and feelings) in a scientifically rigorous way, on the model of the scientific study of human consciousness.

The field faces significant methodological challenges because non-human subjects cannot verbally report their experiences [2]. But if you think the absence of verbal report precludes any scientific investigation of animal consciousness, you should be prepared to say the same about consciousness in preverbal infants and patients in a minimally conscious state. Animal consciousness research rests on the idea that, by synthesising the insights and methods of multiple disciplines, and by identifying a battery of behavioural, cognitive, and neuronal criteria for attributing conscious states, these challenges may be overcome.

## Are Some Animals ‘More Conscious’ Than Others?

At present, the field is young and beset by foundational controversy: controversy about the criteria for consciousness and the methods for studying those criteria [3–7]. At the heart of these debates lies a conceptual question: How can we make sense of variation in consciousness across the animal kingdom? Does it make sense to say that some animals are ‘more conscious’ than others? Does consciousness come in degrees? If it does, how can ‘degrees of consciousness’ be measured and investigated? For example, could a bird be more conscious than a fish? Could an octopus be more conscious than a bee?

In studies of disorders of consciousness in humans, the idea of ‘levels of consciousness’ has been influential [8]. Clinicians assessing patients with disorders of consciousness assign a level of consciousness, with coma at one end of the scale, conscious wakefulness at the other, and various intermediate grades (such as deep sleep and light sleep) in between. It is tempting to apply this to non-human animals. We could attempt to construct a single sliding scale of animal consciousness, along which birds (such as corvids), fish, cephalopods (such as octopuses), bees, and so on could all be placed.

This, however, would be a mistake. Recently, the value of the ‘levels of consciousness’ framework for conceptualising disorders of consciousness in humans has been called into question [9–13]. The main concern is that, if we try to force states of consciousness into a one- or twodimensional scale, we will inevitably neglect important dimensions of variation. Critics of the ‘levels’ framework argue that we should instead adopt a multidimensional framework, capturing several different dimensions of variation.

This point carries over to the case of animal consciousness, where the variation is likely to be even more substantial and multifaceted. If the overall conscious states of humans with disorders of consciousness vary along multiple dimensions, we should also expect the typical, healthy conscious states of animals of different species to vary along many dimensions. If we ask ‘Is a human more conscious than an octopus?’, the question barely makes sense. Any single scale for evaluating questions such as these would end up neglecting important dimensions of variation. For this reason, we suggest that animal consciousness research should adopt a multidimensional approach, not a single-scale approach, when thinking about variation across the animal kingdom.

**Figure 1:**
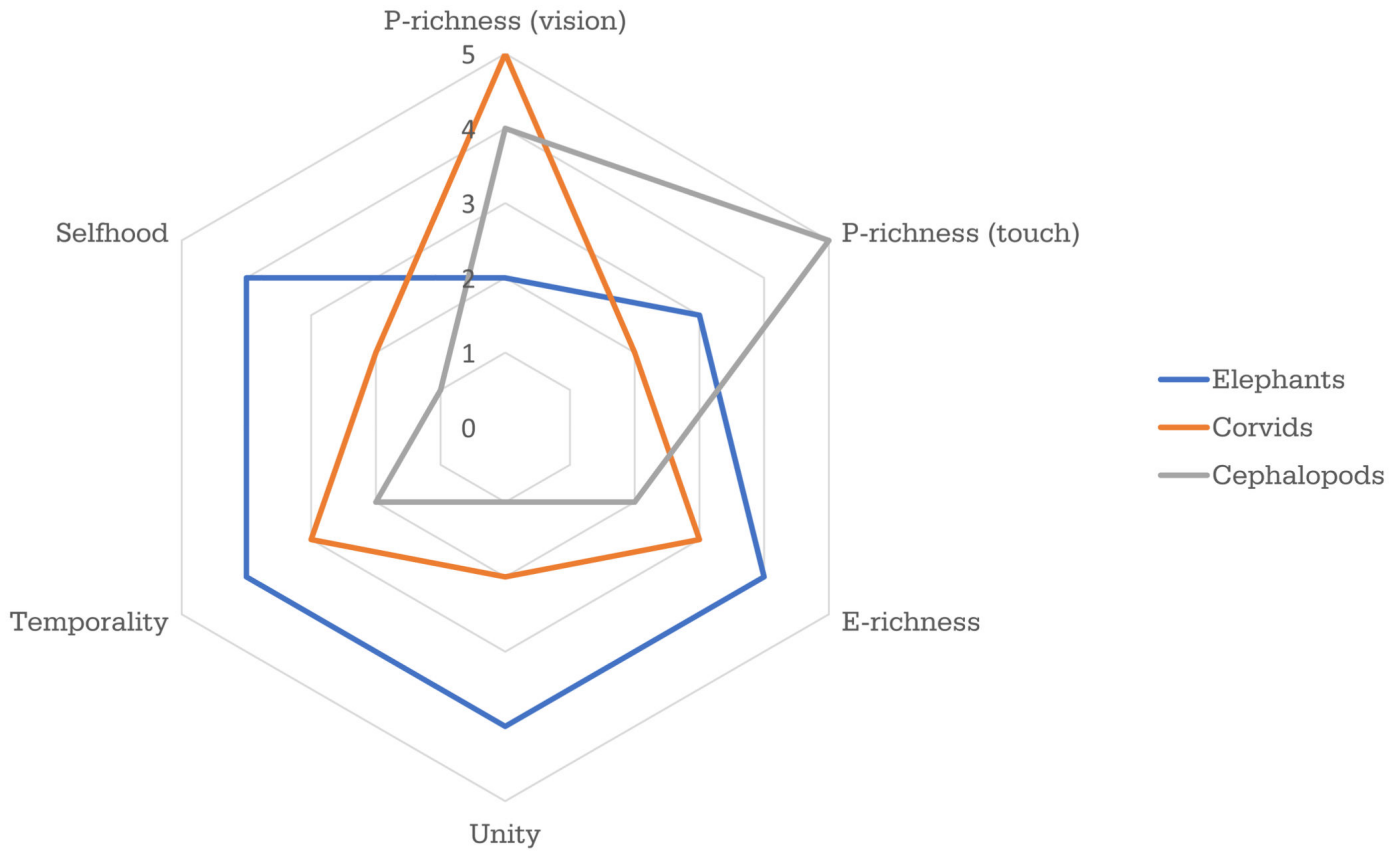
**Key Figure** Hypothetical Consciousness Profiles for Elephants, Corvids, and Cephalopods These hypothetical profiles highlight six important dimensions of variation, with p-richness represented separately forvision and touch. These are not finished, evidence-based profiles: they are conjectures based on current evidence. A key goal for animal consciousness research should be to produce a much richer evidence base for the construction of consciousness profiles and more precise ways of measuring the dimensions. Abbreviations: p-richness, perceptual richness; e-richness, evaluative richness.

What are the main dimensions of variation we can investigate? What do we currently know about those dimensions? What future work would help us learn more about them? Our aim here is to propose a multidimensional framework for thinking about animal consciousness. We will highlight five significant dimensions of variation: perceptual richness (p-richness), evaluative richness (e-richness), integration at a time (unity) and across time (temporality), and self-consciousness (selfhood). For each dimension, we will briefly review existing evidence that bears on that dimension and we will propose future work that could help us rank a given species along that dimension (Figure 1, Key Figure). We will then consider some of the challenges for a multidimensional framework. We turn now to our five dimensions.

## P-Richness

Our first dimension of variation is p-richness (the ‘p’ stands for ‘perceptual’). Animals vary in the level of detail with which they consciously perceive aspects of their environment. Animals that make fine-grained conscious discriminations in a particular sense modality (e.g., vision) can be said to have p-rich experiences in that modality. Any measure of p-richness is specific to a sense modality, so we should not refer to a species’ overall level of p-richness. A species might have richer perceptual experiences than another in one modality, but less rich experiences in a different modality. For example, given their sensory abilities, elephants are likely to have much richer olfactory experiences than humans but less richly detailed visual experiences [14–16].

Within a given sense modality, it is possible to resolve p-richness into different components. For example, the richness of visual experience depends on bandwidth (the amount of visual content experienced at any given time), acuity (the number of just-noticeable differences to which the animal is sensitive), and categorisation power (the animal’s capacity to sort perceptual properties into high-level categories). Does this make it impossible to develop overall evaluations of p-richness for conscious vision? Not necessarily. If one species outperforms another with respect to all three components, it has richer visual experiences overall. However, if the different components of p-richness are poorly correlated (e.g., because some species have low bandwidth and high acuity, or vice versa), we may decide that cross-species comparisons should use these finer-grained dimensions rather than p-richness. That is an issue for further investigation.

To probe questions of p-richness rigorously, we need a way of disentangling conscious and unconscious perception. Blindsight illustrates the difference: subjects report blindness in part of their visual field, but they are able to use visual information about objects in that region to guide action [17–19]. The standard interpretation of blindsight is that the subject has no conscious experience of what they perceive in the blind region. In the absence of verbal report, what provides evidence that a particular stimulus is perceived consciously rather than unconsciously?

In broad terms, there is a neurological route and a cognitive route to evidence of conscious perception. The neurological route involves experimentally induced blindsight. Monkeys with lesions of the primary visual cortex, V1, have been shown to respond like humans with blindsight.

When trained to report the presence or absence of a visual stimulus, they report its absence in a region of their visual field, but they can still use information about that stimulus to guide action in forced-choice tasks [19]. This leads to the following thought: if a stimulus is processed in a brain region such that damage to that region results in blindsight, then a healthy, blindsight-free animal of the species in question probably perceives that stimulus consciously. In principle, this strategy could be extended to non-mammals, based on identifying homologues or analogues of V1 in those animals. While blindsight solely concerns vision, there is some evidence for parallel phenomena in hearing and olfaction [20,21]. The drawback to this neurological route is that it is invasive and difficult.

The cognitive route involves looking for cognitive tasks that are linked to conscious perception in humans and then testing how well the target species of animal performs those tasks when the stimuli are presented in a particular modality. Various forms of learning have been linked to conscious perception [22,23]. One important example is trace conditioning, a version of classical conditioning in which the conditioned and unconditioned stimuli are separated in time. For instance, a tone may be followed, a second later, by a blast of air in your eye. There is evidence that humans learn the association between the tone and the blast only if they consciously experience the stimuli and the temporal relation between them [24,25]. This points to a possible link between conscious perception and the learning of temporal relations. If we find that an animal is able to do trace conditioning on some stimulus, then that is some evidence, albeit not conclusive evidence, that it consciously perceives that stimulus [26,27]. This cognitive route is likely to be easier and cheaper to apply to a wide range of animals. Whichever route we take, one crucial challenge is to design tests that push an animal’s conscious perception to the limit, inducing maximally p-rich experiences. To achieve this, the stimuli need to be carefully tailored to the animal’s sensory abilities and ecology.

## E-Richness

The second dimension of variation is e-richness (the ‘e’ stands for ‘evaluative’). Some conscious emotions, such as pain, fear, grief, and anxiety, feel bad. These are affective experiences with negative valence. Others, such as pleasure, joy, comfort, and love, feel good. These are affective experiences with positive valence. All affective responses have positive or negative valence. Valence provides ‘an evaluative “common currency” for use in affectively-based decision making’ [28]. Animals are likely to vary in the richness of their experiences of valence. We think valence is likely to prove a particularly useful concept for understanding variation because, while it may be dubious to attribute specific human emotions (such as anxiety and grief) to a wide range of animals, valence must be present wherever there is affect-based decision making. Some human emotions, such as thirst, hunger, and pain, are plausibly shared by a wide range of animals [29], but we do not want to take this for granted.

Finding out how positive and negative valence are produced in an animal, and how these processes vary across taxa, should be a central goal of animal consciousness research. Assuming that all conscious animals have an evaluation system of one sort or another, there remains room for grades of sophistication. Some animals may be constantly evaluating small changes in their internal states and external surroundings, as we do, whereas others may respond only to more substantial changes. Like p-richness, e-richness has more than one component. Rich affect-based decision making takes many inputs into account at once (evaluative bandwidth) and is sensitive to small differences in those inputs (evaluative acuity). If these components turn out to be poorly correlated, we may decide that cross-species comparisons need to use finer-grained dimensions.

Experiments probing motivational trade-offs can provide insight into how evaluation systems vary. In one such experiment, rats (*Rattus norvegicus domestica*) were presented with an opportunity to access a sugar solution by entering a cold chamber [30]. The rats traded off the sugar content of the solution against the temperature of the chamber: all else being equal, they were willing to withstand colder temperatures to get sweeter rewards. This is evidence of an evaluative common currency: the value of sugar is weighed subtly against the disvalue of cold. Is it also evidence of conscious experience of the currency? It is relevant that the trade-off is crossmodal: this is not an animal evaluating options using information from a single sense, but an animal weighing the taste of a liquid against the temperature of the ambient environment. This requires the crossmodal integration of information, which has often been linked to consciousness, although it may not strictly require it [31,32].

Similar experiments have been done on iguanas (*Iguana iguana*) [33] and hermit crabs (*Pagurus bernhardus*) [34,35], with similar results. What is lacking, so far, is interspecies comparisons of the sophistication of the evaluations being made by different species and investigations of how sophisticated the evaluations need to be to indicate conscious affect. The motivational tradeoff paradigm has great potential and should be a priority for future work.

## Integration at a Time (Unity)

Conscious experience in healthy adult humans is highly unified. You have a single perspective on the world and everything of which you are consciously aware is part of that perspective. All the experiences generated by your brain have a common subject. There are not two or more subjects housed within the same skull.

Psychologists have long been fascinated by pathologies, such as the split-brain syndrome, in which this unity apparently breaks down [36,37]. Subjects who have had the corpus callosum wholly or partially severed sometimes display disunified behaviour when different stimuli are presented to the two halves of the visual field. If these subjects are asked to verbally describe what they see, they will report what is visible on the right-hand side of their visual field. This is because language is predominantly controlled by the brain’s left hemisphere, which only has access to visual information from the right-hand side. Yet, when asked to draw with the left hand what they see, they will draw what is visible on the left-hand side of the visual field. This is because the left hand is predominantly controlled by the right hemisphere, which only has access to visual information from the left-hand side. This disunity of behaviour leads to a debate about whether experience itself is also disunified. Could there be two subjects within one skull? [36–39].

The same questions can be asked of non-human animals. Birds are particularly interesting in this respect because they are natural split-brains. They have no structure akin to the corpus callosum connecting the two hemispheres of the dorsal pallium, which is homologous to the cortex in mammals [40]. Could every bird be a pair of conscious subjects, intimately cooperating with each other? A similar debate arises with respect to the cerebral ganglia and brachial plexus (a nerve ring around the top of the arms) of the octopus [23,41–43]. These structures are connected, but they have some degree of functional autonomy from each other. Could an octopus have two, or even nine, conscious perspectives on the world? Current evidence does not settle these questions; our aim is only to raise them.

What provides evidence that an animal has a single, unified perspective as opposed to multiple perspectives? It is crucial to investigate cognition as well as neuroanatomy. Here we can draw inspiration from experiments on split-brain humans. One paradigm involves training an animal to perform a task in response to a stimulus presented to one eye and seeing whether the task can still be performed when the stimulus is presented to the other eye: interocular transfer. In pigeons (*Columbia livia*), the visual field for each eye can be divided into two regions: the red field, which is the lower frontal region important for guiding pecking, and the yellow field, which covers the upper frontal and lateral regions. There can be interocular transfer between the red fields of each eye, but there seems to be no interocular transfer between the yellow fields in nearly all individuals [44]. Some particular individuals can do it, but no one knows why [44].

There is a need for more experiments that investigate the integration of the two visual hemifields and the related question of whether information presented to one hemisphere is accessible for the guidance of actions controlled by the other. For cephalopods, there is a further question about the extent to which information presented to the arms is accessible to the brain, or vice versa. The existing evidence in corvids and cephalopods presents a complicated picture, with some studies pointing towards surprising dissociations and others indicating substantial integration (Boxes 1 and 2).

Clues regarding the unity of consciousness may also come from unihemispheric sleep. If one hemisphere sleeps while the other is awake, that is suggestive of more than one stream of consciousness, though not conclusive. This has been observed not only in various birds, but also in dolphins and seals [45].

## Integration across Time (Temporality)

Normal human experience is highly integrated across time. Our experience of the world takes the form of a continuous stream, one moment flowing into the next [46,47]. For example, we experience the leaves of a tree blowing in the wind; we do not infer the motion from a series of static snapshots. Human experience is also temporally integrated across longer timescales. We are able to recall past experiences and simulate future experiences, a form of ‘mental time travel’ [48]. Let us call this dimension temporality.

What could constitute evidence for a temporally integrated stream, rather than a staccato series of fragmented experiences? One possibility is to look for mechanisms that edit sensory input to produce a coherent, continuous stream from discontinuous stimuli. In humans, evidence for such mechanisms comes from the colour-phi illusion, in which two spatially separated, differently coloured dots flashed in sequence are perceived as a single moving dot that changes colour halfway across the gap [49]. The brain is not simply mistaking two static stimuli for a moving stimulus: it is constructing a coherent account of how the stimulus is changing. Colour-phi has received a great deal of discussion in the philosophy of consciousness [50]. What matters here is simply that, if we found colour-phi in non-human animals, this would be evidence that they too have mechanisms that transform a series of discrete stimuli into a coherent experience of change. Although our evidence of colour-phi in humans comes from verbal report, it is possible in principle to study colour-phi in the absence of verbal criteria [51]. Animals could be trained to respond differently to perceptions of continuous and discrete stimuli and to stimuli that change colour half-way and stimuli that do not. We could then present them with a colour-phi test stimulus, gradually reducing the interstimulus interval. Would there be a threshold at which the animal switched from categorising the stimulus as discrete to categorising it as continuous and would the animal categorise the stimulus as one that changes colour half-way?

Turning to integration over longer timescales, what provides evidence for conscious mental time travel? This higher grade of temporality probably requires substantial cognitive sophistication. The most promising places to look for it are great apes, cetaceans, corvids (Box 1), and cephalopods (Box 2). The evidence for mental time travel in corvids is particularly strong. Corvids are able to produce rich and flexible representations of past events and prepare for specific future scenarios [52]. For example, California scrub-jays (*Aphelocoma californica*) plan ahead when making decisions about where to store food [53], and there is evidence that ravens (*Corvus corax*) plan ahead when offered tools or tokens that they will need later for a task that they would never have encountered in the wild [54]. There is evidence that some corvids can plan spontaneously, and such future-oriented behaviours cannot be solely explained through reinforcement learning [53]. Although these results have been criticised [55,56], we regard spontaneous planning as a promising nonverbal indicator of conscious temporal integration.

There is a need for more evidence that planning and remembering in animals involves conscious simulation. What form could this evidence take? If an animal can remember the source of a memory (e.g., vision or smell) and not just the content, that is suggestive of conscious recall, though not conclusive [57]. In humans, a simulated episode (for example, walking across a room) unfolds over the same length of time as a perceptual experience of the same episode [58,59]. If we found evidence that the mental rehearsal of future actions unfolds over the same length of time as the actions themselves, this would be evidence of something strikingly close to human mental time travel.

## Self-Consciousness (Selfhood)

Self-consciousness, or selfhood, is the conscious awareness of oneself as distinct from the world outside. Like all the other dimensions, this is a capacity that admits of gradations [60,61]. A minimal level of self-consciousness may be present in a wide range of animals. It involves registering a difference between self and other: registering some experiences as representing internal bodily events and other experiences as representing events in an external world. Any complex, actively mobile animal needs a way of disentangling changes to its sensory input that are due to its own movements from changes due to events in the world [43,62–64].

A more sophisticated grade of self-consciousness involves awareness of one’s own body as a persisting object that exists in the world [65]. This capacity is plausibly needed to pass a mirror-mark test, in which the test subject is able to recognise a mark seen in a mirror as a mark on its own body. Chimpanzees (Pan *troglodytes*) [66], bottlenose dolphins (*Tursiops truncatus*) [67], Asian elephants (*Elephas maximus*) [68], and magpies (*Pica pica*) [69] have reportedly passed such a test. A striking study in 2019 reported that a fish, the cleaner wrasse (*Labroides dimidiatus),* can also pass the test [70]. Fish able to view a coloured mark on their throat in the mirror were much more likely to exhibit throat-scraping behaviour, as if to remove a parasite, than fish who had transparent marks or no access to a mirror. These results are controversial [71], but they suggest that the grade of self-consciousness required to pass the mirror-mark test is possessed by a wide range of animals.

This falls short of a yet more sophisticated grade of self-consciousness, which involves awareness of oneself as the persisting subject of a stream of experiences, distinct from other such subjects. This is an ability related to mindreading (or theory-of-mind). It involves turning mindreading inward, to recognise oneself as the subject of mental states. We take this to be a form of metacognition, although the relation between metacognition and mindreading is contested [72]. Humans possess this grade and there is (debated) evidence to suggest that non-human apes [73] and corvids [74,75] possess some mindreading ability. However, there is very little evidence of the ‘turning inward’ of mindreading. Evidence that animals can make experience projections, inferences from what they experience in a particular situation to what others will experience, bears on this question. Such evidence has recently been found in great apes. In a study involving chimpanzees, bonobos (*Pan paniscus*), and orangutans (*Pongo* genus), only apes who had themselves experienced a barrier as opaque were able to infer that others would not see objects on the other side of it [76].

## Challenges for a Multidimensional Framework

A multidimensional approach to animal consciousness faces several key challenges. One is to find dimensions at the right grain of analysis. If our goal were to capture all interesting variation in conscious states, we would never have enough dimensions. We have to be pragmatic. The five dimensions discussed previously are intended as top-level categories that can help us coordinate inquiry into finer-grained variation. As noted in the sections on p-richness and e-richness, there may well be ways of resolving our top-level dimensions into new spaces of further, finer-grained dimensions. We welcome debate on the issue of whether our five dimensions achieve the appropriate grain of analysis.

A second challenge is to make sure the dimensions are distinct enough from each other. What is not required is that the dimensions should be completely uncorrelated. If one dimension is found to correlate positively with another, this is an interesting result, not a problem. For example, it may turn out that temporality is correlated with selfhood, because richer forms of temporal integration enable a species to evolve a higher grade of self-consciousness. This is a hypothesis worthy of further investigation. It does, however, matter that the dimensions are conceptually distinct from each other (that they are not the same thing described in two different ways). We have tried to ensure that our dimensions are conceptually distinct. For example, although temporality and selfhood may be correlated, they are different concepts: it is conceivable that an animal could have a richly temporally integrated stream of experiences without any awareness of itself as the subject of those experiences, and it is conceivable that an animal could have temporally fragmented ‘staccato’ experiences while being aware of itself as the subject of those fragments.

A third challenge is to find dimensions that facilitate informative comparisons between species. We hope it will ultimately be possible to devise a standardised battery of tests that generate a ranking of species on each dimension, but we are not there yet. That is a big challenge for the future (see Table 1 for experimental paradigms with the potential to provide some insight). Our aim has been to present dimensions that allow for fruitful experimental investigation, in the hope of stimulating further discussion about how we might try to rank species along these dimensions.

## Concluding Remarks

Our five dimensions of animal consciousness vary across and within species. Instead of thinking about variation between species in terms of levels of consciousness, we should think about multidimensional consciousness profiles.

We are not yet in a position to construct numerical measures of all five dimensions. What we can do is make broad, evidence-based comparisons. For example, neuroanatomical considerations suggest that conscious experience in mammals (which have a corpus callosum) may be more highly unified than in birds (which do not) and that experience in birds may be more highly unified than in cephalopods. This conjecture may be overturned by more detailed evidence, but it is a starting point. Comparisons of this type can be made along all five dimensions, allowing us to build up consciousness profiles that tell us where a species is most likely to fit in the space of possible forms of experience, given the evidence we currently have (see Figure 1 for a conjectural starting point, intended to illustrate the general idea of a consciousness profile). A summary of the current evidence regarding corvids and cephalopods is given in Boxes 1 and 2.

We are still some way from being able to construct detailed, evidence-based consciousness profiles for a wide range of species (see Outstanding Questions). Our aim here has been to make a case for the value of consciousness profiles in preference to the idea of a single sliding scale on which some animals are considered more or less conscious than others.

## Figures and Tables

**Table 1:** Current Experimental Paradigms for Investigating Dimensions of Animal Consciousness.^a^

Dimension	Experimental paradigm	Question being investigated	Refs
P-richness	Induced blindsight	Can blindsight-like phenomena be induced in the animal through lesions to specific brain regions? If so, what information typically reaches those regions? (Drawback: highly invasive).	[19]
	Discrimination learning	Can the animal learn to respond differently to very slight differences between stimuli (and how small can the differences be)?	[118]
	Reversal learning	When stimulus contingencies are reversed, can the animal rapidly learn that they have been reversed? This is potentially linked to consciousness in humans.	[119,120]
	Trace conditioning	Can the animal still learn stimulus contingencies when the stimuli are separated by a temporal gap? This is potentially linked to consciousness in humans.	[24,27]
E-richness	Motivational trade-off	Does the animal weigh different needs against each other in a ‘common currency’ to make flexible decisions?	[30,33–35]
	Outcome devaluation and revaluation	If the value to the animal of a reward is manifestly changed, will the animal change its behaviour quickly?	[121]
	Cognitive bias	Does the animal respond differently to novel stimuli depending on its affective state?	[122]
	Emotional contagion	Is the animal susceptible to ‘catching’ the emotions of other individuals?	[123]
Unity	Interocular transfer	If the animal is conditioned to respond to a stimulus presented in one visual hemifield, can the same response be elicited by presenting it to the other hemifield?	[44]
	Meta-control	If the two visual hemifields are presented with conflicting information, can the animal resolve the conflict?	[124]
	Crossmodal integration	Can the animal integrate information from different sense modalities (e.g., vision and hearing?)	[125]
	Visuo-spatial bias	Does the animal exhibit visuo-spatial biases in behaviour (e.g., a preference for using a particular eye to guide a particular task?)	[108,126]
	Multitasking	When given two tasks simultaneously (e.g., foraging and watching for predators), does the animal divide the labour between the two hemispheres?	[126]
	Electroencephalograph studies of sleep	Does the animal exhibit unihemispheric or bihemispheric sleep?	[45]
Temporality (timescales <1 s)	Apparent motion	Can the animal respond differently to moving and static images? Can it make inferences from video images to real moving objects and vice versa?	[127]
Temporality (timescales >1 s)	Episodic-like memory	Can the animal simultaneously remember ‘what’, ‘where’, and ‘when’ about a specific past event?	[128]
	Source memory	Can the animal remember information about how a memory was acquired (e.g., by vision or by smell)?	[57]
	Memory integration	Can the animal update old memories with new information?	[90]
	Future planning	Can the animal flexibly and spontaneously plan for a future event, and for future desires, without relying on reinforcement learning?	[129]
Selfhood	Mirror-mark	Does the animal recognise a mark seen in a mirror as a mark on its own body?	[66–70]
	Body awareness	Can the animal recognise the position of its own body as a potential obstacle to success in a task?	[130]
	Experience projection	Can the animal predict how others are likely to behave in a scenario on the basis of a specific past experience it had in the same scenario?	[74,76]

a A list of established experimental paradigms with the potential to provide insight into p-richness, e-richness, unity, temporality, and selfhood. There is continuing debate regarding the implications of these paradigms for questions about conscious experience. Inferences to properties of conscious states will be stronger when based on a battery of convergent experimental results from different paradigms. We restrict our attention here to established paradigms (see the main text for suggestions for future work).

## Highlights

In recent years, debates about animal consciousness have moved on from the question of whether any nonhuman animals are conscious to the questions of which animals are conscious and what form their conscious experiences take.

There is an emerging consensus that current evidence supports attributing some form of consciousness to other mammals, birds, and at least some cephalopod molluscs (octopuses, squid, cuttlefish).

If we try to make sense of variation across the animal kingdomusing a single sliding scale, ranking species as ‘more conscious’ or ‘less conscious’ than others, we will inevitably neglect important dimensions of variation.

There is a need for a multidimensional framework that allows the conscious states of animals to vary continuously along many different dimensions, so that a species has its own distinctive consciousness profile.

## Box 1 Current Evidence and Open Questions: Corvids

P-richness: corvids possess rich colour and UV vision [77,78] with superb motion sensitivity [79]. Their vision supports sophisticated cognitive behaviours. For example, New Caledonian crows possess binocular overlap that supports tool manufacture and tool-use [80]. Other corvid species rely on vision to recognise specific individuals [81] and to observe where other birds have hidden food caches [82]. Their keen auditory abilities allow for communication through a rich array of calls and songs [83].

E-richness: various corvid species exhibit play behaviour, which is often linked to positive emotions. For example, corvids have been observed repeatedly sliding down steep snow-covered inclines on a plastic lid [84]. Behaviours suggestive of negative emotions have also been reported. When exposed to models of dead conspecifics, crows recruit neighbours to the scene [85]. Ravens (Figure IA) show a pessimism bias after being exposed to a conspecific in a negative state, resembling emotional contagion [86]. Future research should focus on whether such behaviours involve experiences of anxiety, fear, stress, or grief.

Unity: corvids process information bilaterally: one brain hemisphere exerts dominant control over specific functions. During spatial memory tasks, the right eye system shows a preference for object-specific cues and the left eye system for spatial cues [87]. New Caledonian crows prefer a specific foot during tool manufacturing/use [88]. There is also evidence of lateralised emotional processing [89]. The implications of lateralization for the unity of consciousness require further investigation.

Temporality: corvids remember the ‘what, where, and when’ of past events (episodic-like memory) and anticipate future scenarios. California scrub-jays discriminately retrieve items based on what they remember they had cached, where they cached it, and when [90]. They show flexibility in the temporal patterns they can learn: they can learn that food is inedible after a short interval but will ‘ripen’ after a long interval [91]. Caching decisions are based on future planning. After experiencing that one room never contained breakfast and one room always offered breakfast, jays spontaneously cached in the room that did not offer breakfast [53].

Selfhood: magpies have passed the mirror-mark test [69]. There is also evidence that corvids recognise that they have different perspectives/desires from others. For example, jays use information from their own experience as a pilferer to make inferences about opportunities for theft by others [74]. Male Eurasian jays (Figure IB) feed their female partner the food she would like to eat, adapting to her changing desires [92]. Further research is required to explore the link between these abilities and self-consciousness.

## Box 2 Current Evidence and Open Questions: Cephalopods

P-richness: cephalopods possess rich visual and chemo-tactile perception. Although colour-blind [93,94], they are sensitive to polarised light [95] and can discriminate between patterns ofdifferent contrast [96], size, and shape [97]. They dynamically adjusttheir body patternstoawide range of backgrounds, requiring avisual system thatcan process complex scenes [98]. Cephalopods possess chemoreceptors on their suckers [99,100], which are thought to facilitateataste-by- touch ability [101].

E-richness: play, a behaviour linked to positive emotion, has frequently been reported in octopuses (Figure IA) [102,103]. For example, they have been observed manipulating plastic bottles with jets of water, repeating the behaviour when the current brings the bottles back [104]. What about pain? Octopuses and squid possess sensory neurons that encode noxious stimuli [105–107]. Further research is required to assess whether they experience a conscious, pain-like state, but there is a serious risk that they do.

Unity: cephalopods process information bilaterally: specific cognitive functions are processed by either the left or right side ofthe brain [108,109]. For example, cuttlefish (Figure IB) predominantly use their right eye and associated neural structures to adjust the brightness of their camouflage [110]. However, a recent study showed that cuttlefish relyon stereopsis (depth perception, achieved by integrating information from both eyes) when hunting [111], suggesting that visual information from the two eyes is sometimes integrated. Octopuses prefer different arms for different activities and their arms appear to function partly independently of the brain. Each arm has a self-recognition mechanism that prevents interference with other arms [112]. The question ofwhether cephalopods possess multiple conscious perspectives remains open.

Temporality: cuttlefish integrate experiences across timetoguide behaviour. They rememberthe ‘what, where, and when’ of past events (episodic-like memory). They search for prey depending on what they had previously eaten, where they had sourced their previous meal, and how much time had elapsed since that meal [113]. They adjust foraging behaviour by whether, on a previous occasion, they had seen or smelled the prey [57]. They can also use near-future expectations to modify behaviour: if they expect shrimp to be available later, they will eat less crab now [114].

Selfhood: there is no compelling evidence of cephalopods possessing self-recognition [115], but the question calls for systematic exploration. Their sophisticated abilities to camouflage, disguise themselves as inanimate objects [116], and mimic unappetising/venomous animals [117] (or even algae, as in Figure IA), suggest some grasp of how their body appears to others.

## Outstanding Questions

In the absence ofverbal report, what constitutes evidence that a particular stimulus is perceived consciously rather than unconsciously by an animal? Can we develop tests for conscious perception based on cognitive abilities, such as trace conditioning, that are linked to conscious perception in humans?

How does the sophistication and flexibility of affect-based decision making vary across the animal kingdom and how can we measure and quantify the variation? Are there specific types of flexible decision making that indicate conscious affect?

Can we adapt experiments designed to probe the split-brain syndrome in humans to explore the unity of con-sciousness in animals? What can we infer about the unity of consciousness from the extensive lateralization of bird brains? How can we test for the pres-ence of two (or more) conscious per-spectives in an animal?

Which animals (if any) have mechanisms that edit incoming stimuli for coherence and continuity, creating a flowing stream of consciousness? Can we find evidence in animals of illusions, such as the colour-phi phenomenon, that rely on this type of editing?

How can we show that animals are consciously simulating future scenarios and consciously reliving episodic memories? Can we show that, in some cases, the simulation or memory unfolds over the same length of time as the real, sensory experience of the same event would?

How can we go beyond the mirror- mark test to find evidence of higher grades of self-consciousness? How can we test for experience projection in a wider range of animals?

How should we aggregate evidence concerning p-richness, e-richness, unity, temporality, and selfhood to con-struct evidence-based consciousness profiles? Is there a principled way to score animals along these dimensions to allow quantitative comparisons?
